# Reconstitution of an Infectious Human Endogenous Retrovirus

**DOI:** 10.1371/journal.ppat.0030010

**Published:** 2007-01-26

**Authors:** Young Nam Lee, Paul D Bieniasz

**Affiliations:** 1 Aaron Diamond AIDS Research Center, The Rockefeller University, New York, New York, United States of America; 2 Laboratory of Retrovirology, The Rockefeller University, New York, New York, United States of America; The Pennsylvania State University, United States of America

## Abstract

The human genome represents a fossil record of ancient retroviruses that once replicated in the ancestors of contemporary humans. Indeed, approximately 8% of human DNA is composed of sequences that are recognizably retroviral. Despite occasional reports associating human endogenous retrovirus (HERV) expression with human disease, almost all HERV genomes contain obviously inactivating mutations, and none are thought to be capable of replication. Nonetheless, one family of HERVs, namely HERV-K(HML-2), may have replicated in human ancestors less than 1 million years ago. By deriving a consensus sequence, we reconstructed a proviral clone (HERV-K_CON_) that likely resembles the progenitor of HERV-K(HML-2) variants that entered the human genome within the last few million years. We show that HERV-K_CON_ Gag and protease proteins mediate efficient assembly and processing into retrovirus-like particles. Moreover, reporter genes inserted into the HERV-K_CON_ genome and packaged into HERV-K particles are capable of infectious transfer and stable integration in a manner that requires reverse transcription. Additionally, we show that HERV-K_CON_ Env is capable of pseudotyping HIV-1 particles and mediating entry into human and nonhuman cell lines. Furthermore, we show that HERV-K_CON_ is resistant to inhibition by the human retrovirus restriction factors tripartite motif 5α and apolipoprotein B mRNA-editing enzyme, catalytic polypeptide-like (APOBEC) 3G but is inhibited by APOBEC 3F. Overall, the resurrection of this extinct infectious agent in a functional form from molecular fossils should enable studies of the molecular virology and pathogenic potential of this ancient human retrovirus.

## Author Summary

Retrovirus genomes integrate into the genomes of host cells. If the target cells of a particular retrovirus include germ-line cells, e.g., sperm or egg cells, then retroviral genomes can be inherited like cellular genes. So-called “endogenous” retroviruses have accumulated throughout evolution in the genomes of many organisms, including humans. While all known endogenous retroviruses of modern humans are unable to replicate as retroviruses, the human genome represents a fossil record of ancient retroviruses that once infected our ancestors. In this study, a collection of “dead” endogenous retroviral genomes in modern human DNA was used to deduce the approximate sequence of an ancestral retrovirus, human endogenous retrovirus (HERV)-K, that is now thought to be extinct. A pseudo-ancestral HERV-K DNA sequence was synthesized and used to produce viral proteins and RNA that could reconstitute the HERV-K replication cycle. Thus, the replication and biology of a once-extinct retrovirus can now be studied in the laboratory. Interestingly, reconstituted HERV-K replication experiments, and comparison of the reconstituted HERV-K DNA sequence with the dead HERV-Ks in modern human DNA, suggests that HERV-K may have been extinguished in humans in part by host defenses that induce mutation of retroviral DNA and that the reconstitution of the pseudo-ancestral HERV-K reversed these changes.

## Introduction

A characteristic that is unique to retroviruses is their propensity to integrate their genomes into host-cell DNA as an essential part of their replication cycle. Thus, if the target cell population of a given retrovirus includes germ cells or their progenitors, retroviral genomes can be inherited in a Mendelian manner as so-called “endogenous” forms (see [1] for review). Indeed, endogenous retroviruses have accumulated over time in the genomes of many organisms and are extraordinarily common in mammals, comprising approximately 8% of human DNA [2]. Nonetheless, while some avian, murine, and primate species harbor replication-competent retroviruses within their genomes, intact retroviruses are relatively infrequent and almost all endogenous retroviruses are obviously defective due to the presence of stop codons and frameshifts in one or more genes.

Among the numerous families of defective human endogenous retroviruses (HERVs) found in modern human DNA, the human mouse mammary tumor virus–like 2 (HML-2) subfamily of HERV-K proviruses is of special interest. Even though replication-competent forms of HERV-K(HML-2) have not been found, some proviruses were deposited in the human genome after speciation and represent some of the youngest HERVs known [3–6]. Also, occasional reports link their expression with human disease [7]. The age of an endogenous provirus can be roughly estimated by comparing sequence of the two long terminal repeats (LTRs). At integration, the two proviral LTRs should be identical, but during host DNA replication, each LTR independently accumulates mutations as a function of age, and it is estimated that one difference between two LTRs should occur every approximately 200,000 to 450,000 y. Several HERV-K(HML-2) proviruses have been identified in human DNA that have less than five differences between the two LTRs, suggesting deposition perhaps less than 1 million y ago [3–6]. HERV-K(HML-2)–related proviruses are found only in Old World primates genomes, and many are unique to humans, with nonhuman primate genomes containing empty preintegration sites at orthologous loci. Compellingly, polymorphism exists in humans with respect to the presence or absence of proviruses at some HERV-K integration sites, indicating insertion relatively recently in human evolution [3–6]. Furthermore, many of the younger HERV-K(HML-2) proviruses contain a subset of open reading frames (ORFs) with a few or no mutations [3,6,8]. However, all known HERV-K proviruses are replication defective.

There are several ways in which a defective provirus can proliferate in a host's genome, including via exogenous infection events following complementation in *trans,* where functional proteins are supplied by other endogenous or exogenous viruses. Alternatively, for some retroelements, envelope-independent retrotransposition can occur in *cis,* where an element copies itself and inserts into a new genomic locus within the same cell, forgoing the normal extracellular phase of the retroviral life cycle. Defective proviruses can also be proliferated as a result of long interspersed element retrotransposition [9]. However, most HERV-K(HML-2) replication appears to have been a consequence of autonomous infection by extracellular virions [10,11]. This conclusion is based on the comparatively low number of stop codons and ratio of nonsynonymous to synonymous changes *(dN/dS)* in HERV-K ORFs, indicating a purifying selection on all proteins. Notably, this finding holds for HERV-K Env [10], which should be required for replication that includes an extracellular step but not for any other mode of provirus proliferation.

Ancient retroviruses are of interest, in part because they likely imposed selective pressure on host defenses in human ancestors. Indeed, the tripartite motif (TRIM) 5α and apolipoprotein B mRNA-editing enzyme, catalytic polypeptide-like (APOBEC) 3 proteins that provide part of the host defense against modern retroviruses have been under positive selection for much of primate evolution [12–16]. As a retrovirus that appears to have replicated in the ancestors of modern Old World monkeys, apes, and humans, HERV-K may be partly responsible for this pressure. Moreover, it is conceivable that HERV-K exists today in an undetected replication-competent form in rare humans [4]. However, no studies of the virology or pathogenic potential of this ancient human virus have been possible because a contemporary, replication-competent HERV-K strain has not been identified and may not exist at all.

Despite some functional degradation due to mutation during deposition or during human DNA replication, HERV-K(HML-2) proviruses that have been deposited in human DNA in the past few million years should be reasonably well preserved and have relatively few inactivating mutations. Indeed, various studies have shown that individual proteins from certain HERV-K proviruses can function in vitro [17–25]. We reasoned that it might be possible to resurrect HERV-K(HML-2) in replication-competent form using proviruses that are thought to have most recently entered the human genome as a template. Therefore, we constructed a HERV-K strain whose genome sequence is a consensus of a subset of HERV-K(HML-2) proviruses. Importantly, we demonstrate that all viral proteins necessary for viral replication encoded by this provirus are functional and that proteins and genomes based on the reconstructed HERV-K(HML-2) viral genome can be used to generate infectious exogenous retrovirus particles.

## Results

### Design and Construction of a Consensus HERV-K Genome (HERV-K_CON_)

Initially, our attempts to construct an infectious HERV-K provirus were based on the sole HERV-K provirus (HERV-K113) that has apparently intact ORFs for all viral proteins [6]. This provirus, believed to be among the youngest human-specific HERV-K proviruses, is present in the genomes of a minority of humans. Unfortunately, construction of Gag, Gag-protease (PR), and Gag-PR-Pol expression plasmids based on HERV-K113 resulted in proteins that were poorly expressed and were inefficiently processed and released as virus-like particles (VLPs) (unpublished data). Thus, because presence of intact ORFs did not necessarily imply intact function, we took an alternative approach. Specifically, we adopted a strategy that was based on the assumption that any inherent replication defects that are encoded within HERV-K(HML-2) proviruses present in contemporary human DNA are either unique to each provirus or shared only by a minority of recently integrated proviruses. If this assumption was correct, then each individual defect should be absent from a sequence representing the consensus of a collection of proviruses, even if each provirus that contributes to the consensus is defective.

**Figure 1:**
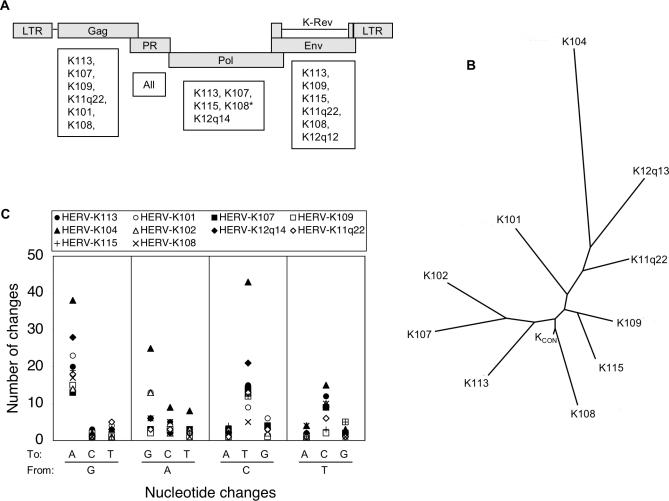
Design and Analysis of a Consensus HERV-K Provirus (A) Diagram of HERV-K(HML-2) provirus. ORFs are depicted as boxes. Proviruses used in the design of HERV-K_CON_ that contain intact versions of Gag, protease, Pol, and Env are listed under each ORF (*K108 encodes a full-length Pol ORF, but a presumed essential YIDD motif is mutated). (B) Phylogenetic analysis of HERV-K_CON_ and the ten proviruses used to generate it, constructed using Kimura 2-parameter algorithm in the TreeMaker program after gap-stripping the sequence alignment (http://www.hiv.lanl.gov/content/hiv-db/CONTAM/TreeMaker/TreeMaker.html). (C) Comparison of HERV-K_CON_ with the ten HERV-K proviruses. Each contributing provirus was compared to HERV-K_CON_ using HYPERMUT, and the number of nucleotide differences for each provirus relative to HERV-K_CON_ is plotted.

We selected a group of ten full-length HERV-K(HML-2) proviruses to derive a consensus HERV-K sequence. Specifically, the ten proviruses with the best scores following BLAST searching of human DNA with full-length HERV-K113 sequence were chosen. As well as HERV-K113 itself, this search yielded HERV-K101, HERV-K102, HERV-K104, HERV-K107, HERV-K108, HERV-K109, HERV-K115, HERV-K11p22, and HERV-K12q13. All of these proviruses are known to be unique to humans, indicating integration into the germ-line within the last 6 million y, when the human lineage is believed to have diverged from the chimpanzee lineage [3–6]. Moreover, several show insertional polymorphism in humans, with intact preintegration sites present in a fraction of the human population, suggesting even more recent replication. While all except HERV-K113 encoded an obvious defect in at least one ORF, all of the selected proviruses also had an intact ORF for at least one of the putative HERV-K proteins (Figure 1A).

The nucleotide encoded by the majority of each of the ten proviruses was deduced for each of 9,472 nucleotide positions to derive HERV-K_CON_. Thereafter, using a set of synthetic, approximately 60 base oligonucleotides spanning the entire HERV-K_CON_ sequence and a PCR- based strategy to progressively link them together, we first constructed a plasmid containing the HERV-K_CON_ proviral genome. The complete proviral consensus sequence and the viral proteins that it encodes are shown in Figure S1. As expected, the HERV-K_CON_ sequence was positioned close to the root of a phylogenetic tree constructed using HERV-K_CON_ itself and each of the ten proviruses used to derive it (Figure 1B). Thus, we reasoned that HERV-K_CON_ represented a reasonable approximation to the ancestor of HERV-K sequences that integrated into the human genome within the past few million years and might, therefore, be capable of replication. Interestingly, pairwise comparisons indicated that the majority of nucleotide differences between HERV-K_CON_ and each of the ten contributing proviruses were either G-to-A or C-to-T changes, or vice versa (Figure 1C). This finding hints at a possible role of cytidine deaminases in driving HERV-K evolution in humans, and perhaps contributing to the inactivation of contemporary proviruses.

### Particle Assembly and Release by HERV-K_CON_ Gag, Gag-PR, and Gag-PR-Pol Proteins

To determine whether the major HERV-K_CON_ structural proteins and enzymes were capable of assembling into retrovirus-like particles, we constructed plasmids expressing the consensus Gag, Gag-PR and Gag-PR-Pol ORFs. The HERV-K genome has an unusual nucleotide composition in that it is relatively A-rich. This feature, which is also characteristic of lentiviruses such as HIV-1, is partly responsible for the nuclear retention of HIV-1 mRNAs and contributes to the requirement for Rev in mediating export of incompletely spliced HIV-1 transcripts. Indeed, HERV-K encodes a functional ortholog of the Rev protein, termed K-Rev or Rec, that mediates nuclear export of HERV-K RNAs [18–20,26]. Therefore, because of the likely requirement for a Rev-like post-transcriptional activator for efficient HERV-K mRNA export, cDNAs encoding the HERV-K_CON_ structural proteins were inserted into a previously described expression vector, termed pCRV1 [27], that provides an HIV-1 Rev response element to the expressed mRNA in *cis* and the HIV-1 Rev protein in *trans*.

**Figure 2:**
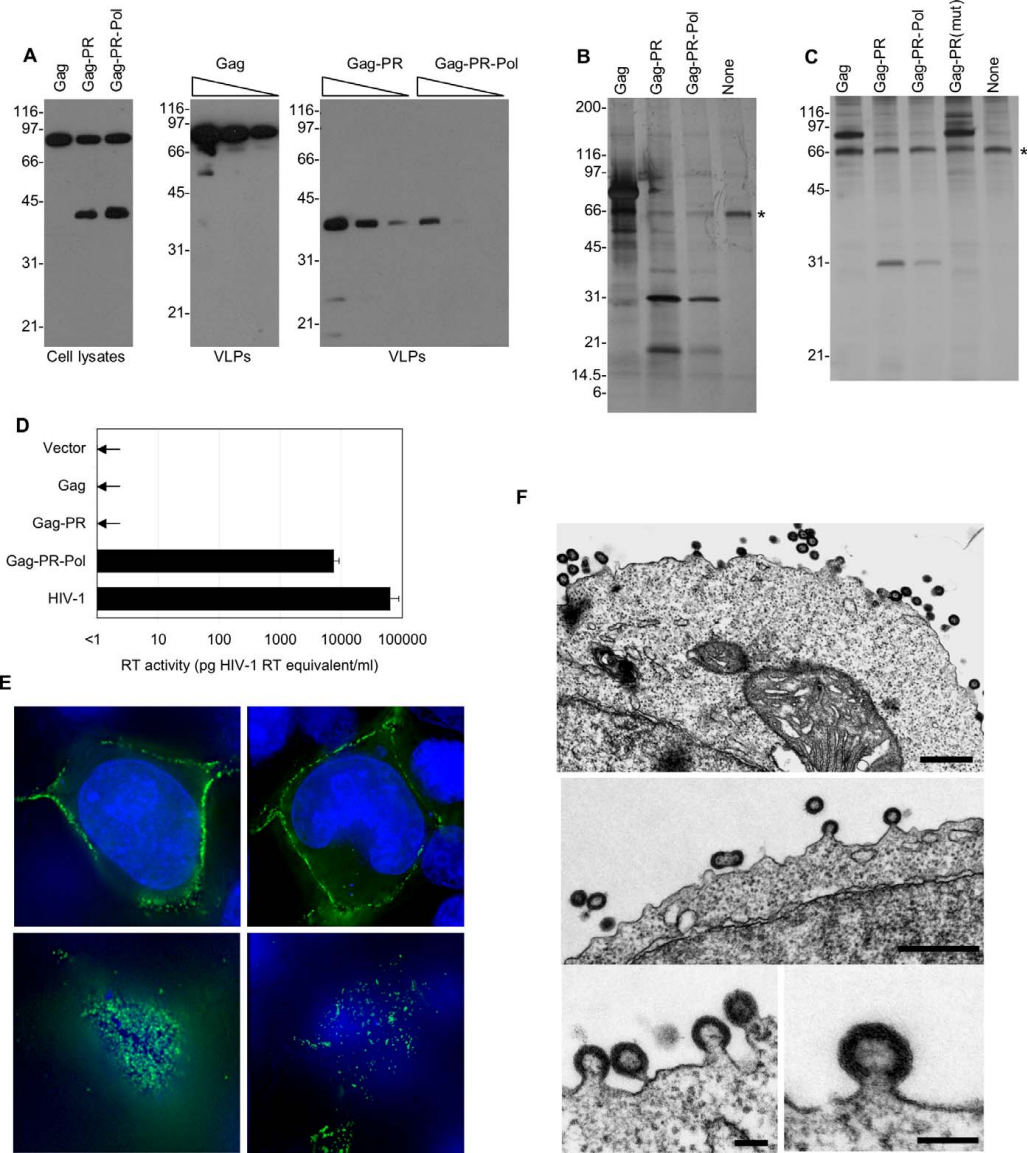
Assembly, Processing, and Release of HERV-K Virus-Like Particles (A–D) 293T cells were transfected with Gag-, Gag-PR–, or Gag-PR-Pol–expressing vectors. (A) Western blot analysis of cell lysates (left) and virions (center and right) using a commercially available antibody to HERV-K Gag. Center shows VLPs from 293T cells transfected with a plasmid-expressing Gag, and right shows VLPs from Gag-PR– and Gag-PR-Pol–expressing 293T cells. Decreasing amounts of virion lysate (0.1, 0.05, or 0.025 μl for Gag; 0.4, 0.2, or 0.1 μl for Gag-PR and Gag-PR-Pol) were loaded to semiquantitatively estimate relative levels of VLP production. (B) Silver stain analysis of a 4% to 20% gradient SDS-PAGE gel loaded with VLPs harvested from 293T cells transfected with plasmids expressing Gag, Gag-PR, Gag-PR-Pol, or empty plasmid control. An asterisk marks a nonspecific 66-kDa protein band, most probably BSA, that is abundant in the culture medium. (C) Silver stain analysis of VLPs harvested from 293T cells containing Gag, Gag-PR, Gag-PR-Pol, or Gag-PR(mut) encoding an active site mutation (DTG-AAA) in protease. An asterisk marks a nonspecific 66-kDa protein band, most probably BSA, that is abundant in the culture medium. (D) Reverse transcriptase activity in culture supernatants of 293T cells transfected with empty pCRV1 (vector) or vectors expressing HERV-K_CON_ Gag, Gag-PR, or Gag-PR-Pol proteins, as indicated. Enzymatic activity was determined relative to a recombinant HIV-1 reverse transcriptase standard and is representative of three experiments. Supernatants from 293T cells transfected with an HIV-1–based proviral plasmid are included for comparison. (E) Two representative 293T cells transfected with HERV-K_CON_ Gag and Gag-GFP expression plasmids. Cells were fixed 18 h post-transfection, and nuclei were stained with DAPI (blue) prior to visualization by deconvolution microscopy. Top, Images acquired at the mid-section of the cell to show localization of Gag-GFP proteins; bottom, focused on the bottom of the cell to show accumulated VLPs at the cell–coverslip interface. (F) Gallery of electron micrographs of 293T cells transfected with a Gag-PR–expressing plasmid. Black scale bars in the upper and middle panels represent 500 nm, while scale bars in the lower two panels represent 100 nm.

Transfection of pCRV1-based plasmids expressing HERV-K_CON_ Gag, Gag-PR, or Gag-PR-Pol resulted in the expression of a protein of approximately 70 to 80 kDa, detected by Western blotting using a commercially available antibody raised against HERV-K Gag (Figure 2A). This approximated to the size expected (74 kDa) of the intact HERV-K Gag precursor. A concurrent analysis of proteins pelleted from culture supernatant through 20% sucrose revealed that Gag expression alone could efficiently generate extracellular particles (Figure 2A). In addition to the 74-kDa Gag precursor, a protein of approximately 40 kDa that reacted with the HERV-K Gag antibody was detected in lysates of cells transfected with Gag-PR and Gag-PR-Pol expression plasmids. While the precise identity of the 40-kDa protein is unknown, it likely represents a proteolytically processed form of Gag and, therefore, this finding suggested that the HERV-K_CON_ protease was active. Consistent with this notion, Western blot analysis of extracellular particles generated following Gag-PR and Gag-PR-Pol expression did not contain detectable Gag precursor but did contain the 40-kDa apparently processed Gag species (Figure 2A).

Analysis of total protein in extracellular particles by SDS-PAGE and silver staining revealed that HERV-K_CON_ Gag expression alone generated particles composed of a single dominant protein of the size predicted for the HERV-K Gag precursor, as expected (Figure 2B). Particles generated by Gag-PR contained a dominant protein of 30 kDa, which based on previous studies likely represents HERV-K_CON_ capsid (CA) [24,28,29]. A smaller protein or proteins of 20 kDa were also observed in Gag-PR particles, which presumably represents other mature Gag processing product or products such as matrix or nucleocapsid (Figure 2B). Additionally, a protein of 40 kDa that likely corresponded to the 40-kDa band detected by Western blotting was also observed on silver-stained gels. However, the 40-kDa protein was a minor species in Gag-PR particles, and it is therefore possible that this protein represents a partly processed intermediate. HERV-K Gag-PR-Pol expression also yielded particles containing the same apparently processed Gag proteins as those generated by Gag-PR but at slightly lower levels (Figure 2B). The appearance of the 30-kDa putative CA protein on silver-stained gels (Figure 2C) was abolished when three predicted active site residues (Asp-Thr-Gly) in the HERV-K_CON_ protease ORF were mutated to Ala-Ala-Ala. Additionally, a higher-molecular-weight protein, possibly representing the Gag-PR precursor, was observed in particles harvested from cells expressing the mutant Gag-PR protein (Figure 2C). Although their low abundance relative to contaminating extraneous cellular proteins and the lack of available antibodies precluded unambiguous identification of Pol proteins in SDS-PAGE analyses of HERV-K_CON_ VLPs, supernatants of 293T cell cultures transfected with the HERV-K_CON_ Gag-PR-Pol expression plasmid contained quite high levels of reverse transcriptase activity, as detected by an ELISA-based assay designed for the detection of HIV-1 reverse transcriptase (Figure 2D). As controls, no reverse transcriptase activity was detected in cultures transfected with HERV-K_CON_ Gag or Gag-PR expression plasmids.

Coexpression of HERV-K_CON_ Gag and Gag–green fluorescent protein (GFP) fusion proteins in 293T cells revealed that HERV-K_CON_ Gag localized predominantly to the plasma membrane, where numerous fluorescent puncta were observed (Figure 2E). Moreover, electron microscopic examination of 293T cells expressing HERV-K_CON_ Gag-PR revealed the presence of cell-associated retrovirus-like particles and structures that appeared to represent assembly intermediates (Figure 2F). Most particles appeared as 100 to 150 nm, apparently spherical immature virions, with a minority assembled as aberrant particles that appeared as two or more connected, partly assembled, virions. While we did not observe unambiguously mature virions associated with the surface of Gag-PR–expressing cells, it is possible that full maturation, which was clearly indicated by the biochemical analysis of extracellular VLPs (Figure 2B and 2C), occurred only after the completion of particle release from cells. Completely or incompletely assembled particles appeared exclusively at the plasma membrane with a morphology resembling partly assembled alpharetroviruses or gammaretroviruses. Even though betaretroviruses represent HERV-Ks closest exogenous retrovirus relatives, no cytoplasmic, nonenveloped particles, typically observed in betaretroviruses, were found.

### Generation of HERV-K_CON_–Based Infectious Virions

**Figure 3:**
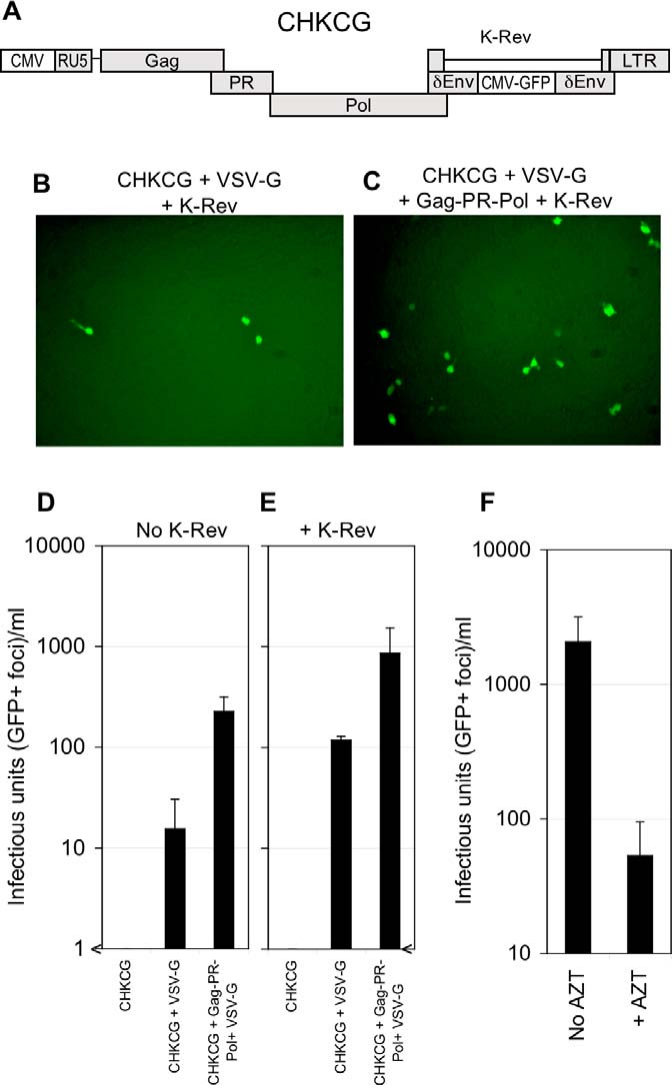
Generation of Single Cycle Infectious Virions Containing HERV-K_CON_ Genomes and Gag-PR-Pol (A) Schematic representation of the HERV-K_CON_–based CHKCG genome. Changes to HERV-K_CON_ are depicted in white boxes. The 5′ LTR was modified to include the CMV promoter inserted in place of the U3 region. The Env ORF, which now contains CMV-GFP, is disrupted. (B and C) Photomicrographs of 293T cell monolayers after infection with CHKCG-carrying HERV-K_CON_(VSV-G) pseudotyped virions, which were generated following transfection of 293T cells with the indicated plasmid mixtures. (D and E) Infectious titers of HERV-K_CON_(VSV-G) pseudotyped virions generated following transfection with the indicated plasmid mixtures in the absence (D) or presence (E) of K-Rev/Rec and using 293T target cells. GFP-positive foci were enumerated visually and expressed as infectious units per milliliter of virion-containing supernatant. (F) Infectious titers of CHKCG containing HERV-K_CON_(VSV-G) using 293T target cells in the presence or absence of 50 μM AZT. All data are representative of at least three experiments.

To determine whether particles containing the HERV-K_CON_ genome, Gag, PR, and Pol proteins were capable of infectious transfer of the HERV-K_CON_ genome to target cells, we inserted a reporter gene cassette (cytomegalovirus [CMV]-GFP) into the *env* gene of the HERV-K_CON_ proviral plasmid. Additionally, because the HERV-K LTR promoter is extremely weak in 293T cells (unpublished data), we replaced U3 sequences 5′ to the TATA box with corresponding sequences from the promoter/enhancer of CMV. This construct was named CHKCG (Figure 3A). As expected, transfection of this Env-defective CHKCG construct in 293T cells resulted in GFP expression in transfected 293T cells, but inoculation of target cells with 0.2-μm filtered supernatant harvested from these cells did not result in infectious transfer of the reporter gene. However, when an envelope protein from vesicular stomatitis virus (VSV-G) was expressed in *trans,* clear GFP expression was observed in rare foci of target cells inoculated with filtered supernatant from CHKCG-transfected cells. Moreover, by boosting HERV-K protein expression, the yield of infectious virions was improved (Figure 3B–3E). Indeed, when K-Rev/Rec was expressed in *trans* with CHKCG and VSV-G, infectious particle yield was in excess of 10^2^ IU/ml (Figure 3B and 3E). Similarly, when the HERV-K_CON_ Gag-PR-Pol expression plasmid was provided in *trans,* the yield of infectious particles also increased to greater than 10^2^ IU/ml (Figure 3D). The combined expression of CHKCG, VSV-G, HERV-K Gag-Pol, and K-Rev/Rec yielded the highest infectious titers (up to 10^3^ IU/ml, Figure 3C and 3E), and this combination of plasmids was used to generate infectious HERV-K_CON_(VSV-G) pseudotyped particles in subsequent studies. While this infectious titer is low compared to that generated by many exogenous retroviruses (e.g., murine leukemia virus [MLV] and HIV-1), the yield of infectious HERV-K particles was of the same order as that obtained with similarly constructed human T-cell lymphotropic virus-I–based vector systems ([30] and unpublished data).

To verify that transfer of reporter gene expression by HERV-K_CON_ particles was via bona fide retrovirus-based transduction, we inoculated 293T cells with HERV-K_CON_(VSV-G) particles containing the CHKCG genome in the presence of azidothymidine (AZT), a reverse transcriptase inhibitor. AZT is a thymidine analog chain terminator and is known to inhibit reverse transcriptases from a wide variety of retroviruses [31]. As can be seen in Figure 3F, application of AZT to target cells inhibited HERV-K–mediated reporter gene transduction by approximately 30-fold. Thus, reporter gene transfer by HERV-K_CON_ was clearly dependent on reverse transcription.

### Transduction by HERV-K_CON_ Results in Bona Fide Retroviral Integration

**Figure 4:**
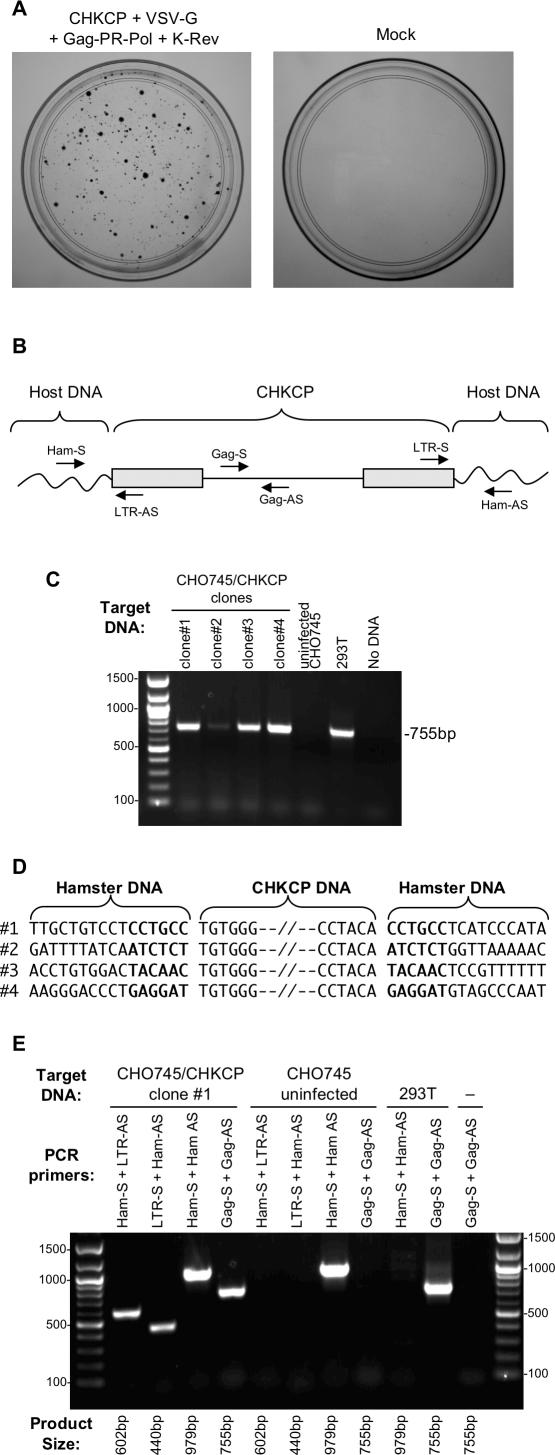
Transduction Mediated by HERV-K_CON_ Gag-PR-Pol and Genomes Results in Stable Proviral Integration (A) Puromycin-resistant colonies of 293T cells infected with either VSV-G pseudotyped (left) or Env defective (right) virions carrying the CHKCP genome. Infected 293T cells were selected in 0.5 μg/ml puromycin for 2 wk and then fixed and stained to reveal colonies of viable cells. Data are representative of at least three experiments. (B) Experimental strategy for detection of HERV-K_CON_ proviruses in CHO745 cells using PCR primers targeted to HERV-K_CON_ Gag and LTR sequences, or flanking hamster DNA sequences. (C) PCR amplification of HERV-K_CON_ *gag* DNA using Gag-S and Gag-AS primers in four expanded clones of puromycin-resistant CHO745 cells transduced with CHKCP-containing HERV-K_CON_(VSV-G) particles. (D) Nucleotide sequences at the 5′ and 3′ ends of integrated CHKCP proviral DNA, revealing six nucleotide duplicated sequences at the CHKCP integration sites. (E) Verification of the presence and absence of an integrated provirus and the empty preintegration site in CHKCP-transduced and naïve CHO745 cells using combinations of HERV-K and hamster DNA targeted PCR primers (see [B] for primer design strategy). DNA templates and PCR primer pairs used are indicated above each lane, and the expected PCR product size is given below each lane. A representative analysis of a single CHKCG-carrying CHO745 cell clone is shown; similar results were obtained with two additional clones. Uninfected CHO745 cells and human 293T cells serve as controls.

In some cases, low levels of reporter gene expression mediated by retroviral gene transfer can be mediated by reverse-transcribed but nonintegrated retroviral DNA, which can exist as linear or circular forms in target cells [32–34]. However, these retroviral DNA forms are diluted during cell division and eventually lost. Stable retrovirus-mediated gene transfer that is transferred to both daughter cells requires that retroviral DNA be integrated into the target cell genome. While the formation of clear multicellular foci of GFP-positive cells suggested the reporter gene was maintained in daughter cells, integration events are most effectively assayed by daughter cell colony formation under antibiotic selection using retroviral genomes that carry resistance markers. Therefore, we constructed a variant of the CHKCG genome (Figure 3A) in which the CMV-GFP cassette was replaced by one carrying a CMV-driven puromycin resistance gene, termed CHKCP. We generated HERV-K_CON_(VSV-G) particles carrying CHKCP in the same way as previously (Figure 3) and found that puromycin-resistant colonies were formed following exposure of 293T target cells to these virions and antibiotic selection (Figure 4A). Indeed, the infectious titers of puromycin resistance transducing particles were similar to that of GFP-transducing particles.

To further demonstrate that HERV-K_CON_ genomes were capable of integration, hamster (CHO745) cells were infected with HERV-K_CON_(VSV-G) particles carrying the CHKCP genome and four single cell clones were derived from the resulting puromycin-resistant cell population. Cellular genomic DNA was extracted following expansion of the clones for 2 wk in culture and analyzed for the presence of integrated HERV-K DNA using a PCR-based strategy (Figure 4B). Hamster CHO745 cells were used for these experiments, because they were found to be as sensitive as human cells to HERV-K_CON_(VSV-G) infection (see below), but unlike human cells, they lack endogenous HERV-K proviruses that would complicate detection and analysis of de novo HERV-K integration events. As can be seen in Figure 4C, PCR analysis using HERV-K *gag* specific PCR primers revealed that each of the CHKCP-transduced clones, but not parental CHO745 cells, carried HERV-K DNA. Next, sequences flanking the integrated provirus were identified using a PCR-based strategy (GenomeWalker kit; Clontech, http://www.clontech.com) and in each case revealed the presence of a six-nucleotide duplicated sequence immediately flanking the provirus (Figure 4D). For three CHKCP-transduced CHO745 cell clones, PCR primers were designed that targeted hamster DNA sequences flanking the integrated HERV-K_CON_ provirus (Figure 4B and 4E), and these were used to authenticate the presence of the intact preintegration site in uninfected hamster cells (e.g., Figure 4E). Moreover, PCRs using combinations of the hamster DNA-specific and HERV-K–specific PCR primers were used to authenticate the presence HERV-K provirus/hamster cellular DNA junctions in three of the CHKCG-transduced clones (e.g., Figure 4E).

Overall, these experiments demonstrate that HERV-K genomes can be replicated via exogenous infection in a reverse transcriptase–dependent manner, resulting in stable and authentic integration into the target cell genome.

### HERV-K_CON_ Tropism

**Figure 5:**
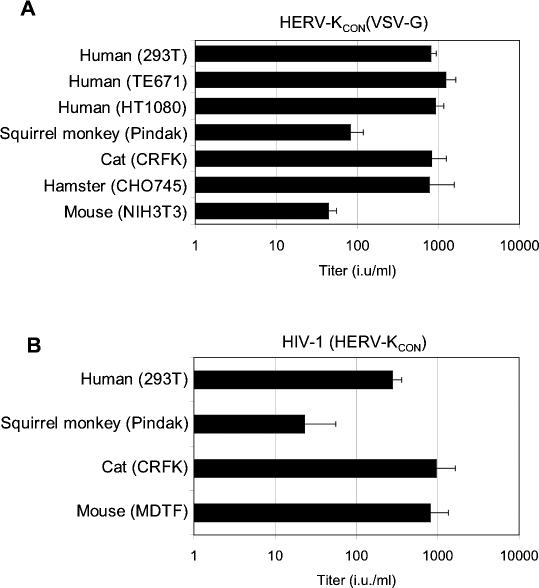
HERV-K_CON_ Tropism (A) Human, squirrel monkey, feline, hamster, or murine cells were infected with VSV-G pseudotyped HERV-K_CON_ particles. Two days postinfection, GFP^+^ foci were quantified microscopically, and titers are expressed as number of infectious units (i.u.) per milliliter of virus-containing supernatant applied. (B) Human, squirrel monkey, feline, or murine cells were infected with HERV-K_CON_ Env pseudotyped HIV-1 particles as in (A). Two days postinfection, GFP-positive foci were quantified. All data are representative of at least three experiments.

Next, we determined whether VSV-G pseudotyped HERV-K_CON_ particles could transduce reporter genes into cells other than 293T and CHO745. As can be seen in Figure 5A, several target cells of human, squirrel monkey, feline, and rodent origin could be infected by HERV-K_CON_(VSV-G). However, it was noticeable that murine NIH3T3 cells and squirrel monkey Pindak cells were somewhat less sensitive to HERV-K_CON_(VSV-G), compared to the human and feline cells. The human cells were each quite similar in their sensitivity to HERV-K_CON_(VSV-G) even though 293T cells display little or no TRIM5α-dependent resistance to retroviruses such as EIAV or N-tropic MLV, while TE671 and HT1080 exhibit strong TRIM5α-dependent resistance to N-tropic MLV and EIAV. This finding suggested that HERV-K_CON_ may not be sensitive to human TRIM5α.

Additionally, to test whether the HERV-K_CON_ envelope sequence was functional, it was inserted into the HIV-1–based expression vector pCRV1 and expressed along with HIV-1 Gag-Pol proteins and the packageable GFP-expressing HIV-1 vector CSGW. This transfection mixture should generate HIV-1 particles, putatively pseudotyped with the HERV-K_CON_ envelope protein. Notably, HIV(HERV-K_CON_) particles were capable of infecting 293T cells, with titers of around 3 × 10^2^ IU/ml (Figure 5B), while particles generated in the absence of HERV-K_CON_ Env were noninfectious. Inoculation of cells from a small panel of mammalian species revealed that several, including those of human, squirrel monkey, murine, and feline origin, could be infected with HIV-1(HERV-K_CON_) pseudovirions (Figure 5B). While attempts were made to generate infectious particles that contained both HERV-K_CON_ cores and Env proteins, we were not able to detect infection events using this combination. Nevertheless, these experiments indicate that the HERV-K_CON_ genome contains all functional components required to complete an exogenous retroviral replication cycle.

### Sensitivity of HERV-K_CON_ to Retrovirus Restriction Factors

**Figure 6:**
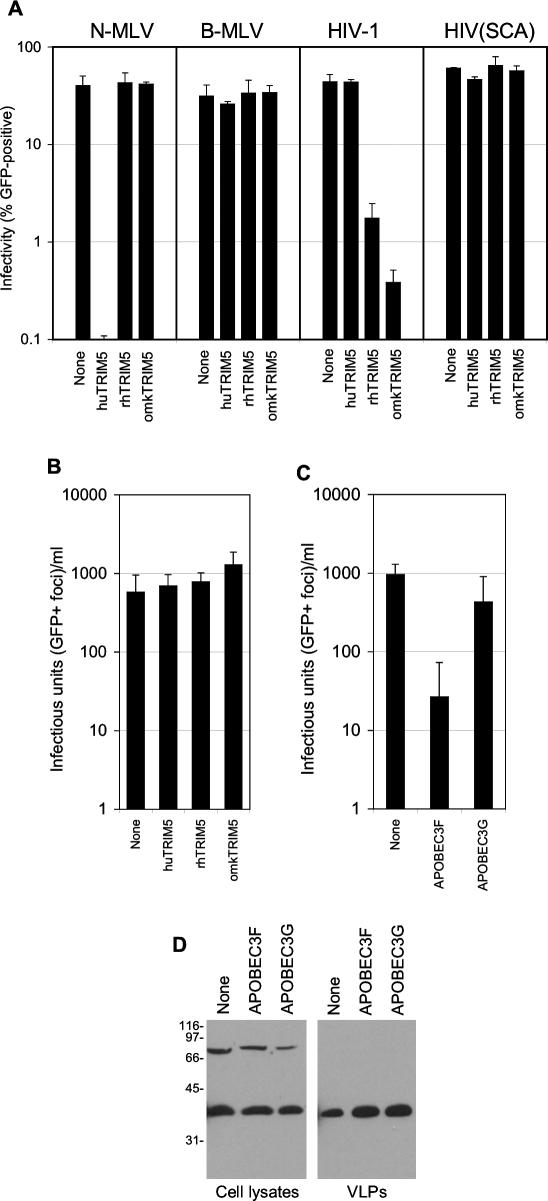
Effects of TRIM5 and APOBEC3 Proteins on HERV-K_CON_ Infectivity (A) Unmanipulated CHO cells or variants stably expressing human TRIM5α, rhesus monkey TRIM5α, or owl monkey TRIM-Cyp were infected with VSV-G pseudotyped retroviral vectors that are sensitive to one or more of the TRIM5 proteins (N-MLV or HIV-1) or TRIM5-resistant controls (B-MLV or HIV-1 carrying SIVmac CA HIV(SCA)), as indicated. Two days postinfection, the percentage of GFP^+^ cells was determined using FACS. (B) The same panel of CHO-derived TRIM5-expressing CHO cell lines were inoculated with HERV-K_CON_(VSV-G). Two days postinfection, GFP^+^ foci were quantified. (C) APOBEC3F and APOBEC3G expression plasmids were cotransfected into 293T cells during generation of CHKCG-containing HERV-K_CON_(VSV-G) particles. Fresh 293T cells were infected with the resulting viral supernatant, and GFP^+^ foci were quantified 2 d later. (D) Western blot analysis, using the anti–HERV-K Gag antibody, of cell and HERV-K_CON_ virion lysates generated upon coexpression of APOBEC3F or APOBEC3G, as indicated. All data are representative of at least three experiments.

To test the sensitivity of HERV-K to retrovirus restriction factors that it might encounter in human cells and might be responsible for attenuation or extinction of replication therein, we first challenged unmodified, or human TRIM5α-expressing, hamster (CHO)-derived cell lines with HERV-K_CON_(VSV-G). Despite the fact that the human TRIM5α-expressing cell line was greater than 100-fold resistant to N-tropic MLV relative to the control cell line or B-tropic MLV (Figure 6A), HERV-K_CON_ infected unmanipulated and human TRIM5α-expressing cells with nearly identical efficiency (Figure 6B). Additionally, CHO cells expressing rhesus macaque TRIM5α or the unique owl monkey variant of TRIM5 (TRIM-Cyp) were also similarly sensitive to HERV-K_CON_(VSV-G) infection as unmanipulated control cells (Figure 6A). This was despite the fact that CHO cells expressing rhesus monkey TRIM5α and owl monkey TRIMCyp were about 30-fold and 100-fold, respectively, resistant to HIV-1 infection compared to HIV-1 carrying an SIV_MAC_ CA (Figure 6A).

Next, we tested whether APOBEC3G and APOBEC3F were capable of inhibiting HERV-K_CON_ replication. These cytidine deaminases are the major inhibitors of Vif-deficient HIV-1 infectivity, although APOBEC3G is a significantly more potent inhibitor of HIV-1 replication than is APOBEC3F. Surprisingly, APOBEC3G expression during particle production only marginally inhibited HERV-K_CON_(VSV-G) infection, while APOBEC3F more potently reduced infectivity, reducing titers by about 50-fold (Figure 6C). This was despite approximately equivalent levels of HERV-K_CON_ Gag expression and generation of viral particles in the presence or absence of APOBEC3G or APOBEC3F (Figure 6D). Overall, of the restriction factors tested that are likely to be encountered in human cells, HERV-K_CON_ appeared to be resistant to human TRIM5α and APOBEC3G proteins but sensitive to APOBEC3F.

## Discussion

Here, we constructed a HERV-K provirus whose sequence resembles that of an ancestral human-specific HERV-K(HML-2). We demonstrate that all viral proteins encoded by this provirus are capable of functioning in the context of a retroviral replication cycle. While some recent studies have reconstituted “live” viruses from synthetic DNA [35,36], this and a similar study of HERV-K which appeared online while this manuscript was in review [37] are the first examples in which the replication cycle of a virus has been reconstituted using a group of sequences that represent ancient fossils and are demonstrably defective. The methods used here are conceptually similar to those applied to the reconstitution of the transposable element Sleeping Beauty, in which a functional Tc1/mariner-type transposon present only in defective forms in fish DNA was reconstituted [38]. Successful reconstitution in that study was achieved using a majority consensus sequence to synthesize an active trasposase protein and selecting *cis*-acting sequences from a representative element that closely resembled those of the majority consensus sequence [38].

HERV-K likely replicated in the ancestors of humans for approximately 30 million y but is not known to exist as a replication-competent virus today. Indeed, it is possible, even likely, that HERV-K has not replicated as a retrovirus for hundreds of thousands of years. It was not obvious what the optimal approach to reconstitute functional HERV-K sequences would be, since variation in HERV-K sequence could arise through natural variation via error-prone reverse transcription, mutational degradation after deposition in the primate germ-line, or cytidine deamination before, during, or after during initial germ-line deposition (see below). Moreover, it was possible that the population of proviruses accessible to us in modern DNA represented a highly biased sample of HERV-K genomes where defects might have been positively selected during primate evolution. Thus, rather than attempt reconstruct the evolutionary history of HERV-K in primates, we adopted a conservative approach to reconstitute functional sequences, selecting ten proviruses that were most similar to a relatively young and comparatively intact HERV-K provirus (HERV-K113), reasoning that these were the least likely to have undergone substantial sequence degradation. Moreover, all of the selected proviruses were unique to human DNA, and some were polymorphic in humans, suggesting comparatively recent replication. While it was possible that all of the selected proviruses would have a common lethal defect, this appeared not to be the case. Indeed, by compiling a simple majority consensus sequence, we successfully removed individual lethal defects represented in the group of proviruses that contribute to the consensus sequence, allowing replication of the consensus genome in a bona fide reverse transcription–dependent manner that resulted in the stable integration of HERV-K_CON_ genomes into target cells. Analysis of the replication cycle of HERV-K_CON_ in human cells allowed preliminary characterization of aspects of HERV-K biology that have heretofore been refractory to investigation.

Assembly of HERV-K virions at the plasma membrane is notable, given that the exogenous retroviruses that are most closely related to HERV-K include mouse mammary tumor virus and Mason-Pfizer monkey virus, both of which are betaretroviruses that assemble complete capsids within the cytoplasm of infected cells. Nonetheless, previous analyses have suggested that the small number of human cell lines that express HERV-K exhibit plasma membrane localized assembly intermediates [28,29], as was observed here for HERV-K_CON_. Moreover, previous work has shown that a single amino acid mutation in MPMV Gag protein can change its assembly characteristics from cytoplasmic to plasma membrane associated assembly [39]. Thus, it should not be surprising that HERV-K assembly appears morphologically different to that of its betaretrovirus relatives.

Two major components of intrinsic defense against retrovirus and retroelement replication in primate cells are the TRIM5 and APOBEC3 gene products [40–42]. Analysis of these genes in modern primates indicates that these genes have likely been under positive selection pressure for significant portions of primate evolution [12–16]. As an endogenous retrovirus that has also apparently replicated exogenously and has been active for much of Old World primate evolutionary history, HERV-K is an excellent candidate for an agent that has imposed sustained evolutionary pressure on antiretroviral defenses present in modern primates. Nonetheless, HERV-K infection was not inhibited by the TRIM5 proteins that were tested. In the case of human TRIM5α, this was not unexpected, because HERV-K_CON_ was derived from human-specific proviruses that must, by definition, have replicated in humans at some point in their evolution and may, therefore, have evolved resistance to human TRIM5α. However, HERV-K_CON_ was also resistant to rhesus monkey TRIM5α and also TRIM-Cyp, a form of TRIM5 that is unique to owl monkeys [43,44], a New World monkey species that does not carry HERV-K. At present, therefore, there is no evidence that TRIM5 proteins and HERV-K have exerted reciprocal evolutionary pressure during primate evolution. However, analysis of CA sequences reconstructed from more ancient groups of HERV-K proviruses and inserted into HERV-K_CON_, as well as inclusion of more TRIM5α variants, may be illuminating. The studies described herein suggest that such approaches to study interactions between ancient retroviruses and their hosts should be feasible.

It was notable that the process of generating a consensus HERV-K genome, in effect, primarily involved the replacement of A and T nucleotides in modern defective proviruses with G and C nucleotides, respectively. The position of the consensus sequence near the root of the phylogenetic tree suggests that a G or C “ancestral” state at many nucleotide positions in human-specific HERV-K genomes has been replaced by A and T nucleotides in modern defective proviruses. While these results do not necessarily lead to the conclusion that cytidine deaminases are responsible for the reduction or extinction of HERV-K replication in humans, they hint that this may have been the case. At a minimum they suggest that cytidine deamination events have impacted HERV-K evolution in humans. While G-to-A changes were the most frequently represented in comparisons of HERV-K_CON_ with contemporary proviruses, plus-strand C-to-T changes also appeared to be overrepresented. While most APOBEC-induced mutation is thought to result from deamination of minus strand cytidines during reverse transcription [45–48], at least some APOBEC proteins are also are capable of inducing C-to-T changes on the plus strand of proviruses, by catalyzing deamination of viral RNA or perhaps dsDNA [49]. HERV-K replication was sensitive to APOBEC3F but only marginally affected by APOBEC3G. This was somewhat surprising and clearly distinguishes HERV-K from Vif-deficient HIV-1 which is more sensitive to APOBEC3G than to APOBEC3F [50–53]. Indeed, an overall view is emerging that a variety of APOBEC3 proteins can be selectively active against various retroelements and retroviruses [54] but HERV-K has yet to be analyzed. Of note is the observation that the patterns of apparent cytidine deamination events in modern HERV-K proviruses do not precisely match those predicted if APOBEC3F was primarily responsible for G-to-A and C-to-T mutation therein. Therefore, further analysis of HERV-K sequences in humans and Old World primates and the impact of their various APOBEC proteins on HERV-K_CON_ replication may illuminate an evolutionary history of ancient host–retrovirus conflicts.

## Materials and Methods

### Derivation, analysis, and synthesis of HERV-K_CON_.

The complete HERV-K113 proviral sequence was used to search human genome sequence using National Center for Biotechnology Information nucleotide-to-nucleotide BLAST. Multiple entries of the same HERV-K proviruses were identified by inspection of flanking genomic sequence and excluded, and the most recently sequenced entries were used for the alignment. The top ten hits were aligned using AlignX program of Vector NTI Advance 10.0.1 (Invitrogen, http://www.invitrogen.com) to derive a consensus sequence that was termed HERV-K_CON_. HERV-K_CON_ sequence was compared to other HERV-K(HML-2) proviruses using HYPERMUT (http://www.hiv.lanl.gov/content/hiv-db/HYPERMUT/hypermut.html) [55].

The complete HERV-K_CON_ proviral sequence was synthesized using overlapping oligonucleotides of approximately 60 bases spanning the entire genome. Oligonucleotides were assigned to 13 groups corresponding to 13 HERV-K_CON_ fragments of approximately 700 nucleotides and assembled using sequential PCRs. In the first round, all oligonucleotides were included in the reaction and 15 cycles of synthesis were executed using Pfu DNA polymerase (94 °C for 10 s, 45 °C for 20 s, 72 °C for 30 s). Thereafter, an aliquot of the reaction product was subjected to amplification using the 5′ and 3′ oligonucleotides in each group (94 °C for 20 s, 45 °C for 20 s, 72 °C for 3 min; 15 cycles). Fragments from regions of the HERV-K genome lacking convenient restriction sites were assembled into longer fragments of up to 1.5 kb via overlap extension PCR. A derivative of the low-copy-number plasmid vector, pXF3, was derived by inserting a synthetic oligonucleotide encoding the restriction sites ClaI, XhoI, EcoRV, SpeI, AgeI, SacI, DraIII, XbaI, and NheI that corresponded to convenient restriction sites in the HERV-K_CON_ genome. These were used to sequentially insert the various synthetic DNA fragments, thereby generating the final pXF3/HERV-K_CON_ proviral plasmid.

### HERV-K_CON_–derived expression plasmids.

CHKCG was created from the pXF3/HERV-K_CON_ proviral plasmid by first replacing HERV-K U3 sequences 5′ to the TATA box with CMV promoter/enhancer sequences using overlapping PCR and ClaI and EcoRV restriction sites to generate pXF3/CMVP HERV-K_CON_. In parallel, an EGFP cDNA (Clontech) was inserted into pCR3.1 (Invitrogen), and then the CMVP-EGFP cassette was PCR amplified and inserted into KpnI site of pXF3/CMVP HERV-K_CON_ to create CHKCG. Similarly, a Puro cDNA was digested from pMSCVPuro (Clontech) with HinDIII and XbaI and inserted into pCR3.1. Thereafter, a CMVP-Puro cassette was PCR amplified and cloned into pXF3/CMVP-HERV-K_CON_ to create the CHKCP genome.

### Other HERV-K expression plasmids.

pCRVI/Gag, pCRVI/Gag-PR, and pCRVI/Gag-PR-Pol were generated by insertion of the respective ORFs from pXF3/HERV-K_CON_ into the NotI restriction site of pCRVI. Similarly, a PCR-amplified HERV-K_CON_ Env-encoding fragment was inserted using EcoRI and NotI restriction sites, generating pCRVI/Env. A mutant form of pCRVI/Gag-PR was generated by substituting the conserved putative active site residues (DTG) to AAA. The two K-Rev/Rec exons were PCR amplified from BAC RP11-33P21 (Invitrogen) containing the HERV-K108 sequence, which happens to encode a K-Rev/Rec protein that is identical to the consensus sequence, joined using overlapping PCR, and inserted into EcoRI and XhoI of pCR3.1 to generate pCR3.1/K-Rev.

### Cell lines and transfection.

The 293T, MDCK, Pindak, TE671, HeLa, CRFK, and HT1080 cells were maintained in DMEM supplemented with 10% fetal calf serum and gentamicin. CHO745 cells and TRIM5-expressing derivatives were cultured in Ham's F-12 medium with the same additives. The 293T cells were transfected in 10-cm plates at 6 × 10^6^ cells per plate or in six-well plates at 1 × 10^6^ cells per well using polyethylenimine as previously described [56]. Medium was changed 24 h after transfection, and virus-containing supernatants were collected after an additional 24 h.

### HERV-K protein analysis.

The 293T cells were transfected with 10 μg of pCRVI/Gag, pCRVI/GagP-PR, pCRVI/Gag-PR-Pol, or pCRVI. Two days after transfection, the supernatant was collected, filtered (0.2 μm), and ultracentrifuged through a 20% sucrose layer at 100,000*g* for 90 min at 4 °C. The pelleted VLPs and corresponding transfected cells were resuspended in SDS-PAGE loading buffer and separated on 10% SDS-PAGE gels (Bio-Rad, http://www.biorad.com). Proteins were transferred onto nitrocellulose membrane and probed with an anti–HERV-K Gag antibody (Austral Biologicals, http://www.australbiologicals.com). Alternatively, VLPs were separated on 4% to 20% gradient or 10% SDS-PAGE gel (Bio-Rad) and silver stained using a kit, as per the manufacturer's instructions (Sigma-Aldrich, http://www.sigmaaldrich.com).

Reverse transcriptase activity in 293T culture supernatants was measured using a commercially available reverse transcriptase assay (Cavidi, http://www.cavidi.se) in which BrdUTP is incorporated into a plate-bound oligo(dT)/poly(rA) substrate. Thereafter, solid phase polymerized BrdU is detected using an anti–BrdU–alkaline phosphatase conjugate and a colorimetric substrate. Activity is standardized using a recombinant HIV-1 RT standard.

### HERV-K_CON_ and HIV-1(HERV-K) pseudotype infection assays.

To generate VSV-G pseudotyped HERV-K_CON_ particles, 293T cells in six-well plates were transfected with 1.3 μg of CHKCG or CHKCP, 1 μg of pCRVI/Gag-PR-Pol, 0.5 μg of pCR3.1/Rec, and 0.2 μg of VSV-G. Empty control vectors were transfected when necessary. In some experiments, 1 μg of plasmids expressing myc-tagged human APOBEC3F and APOBEC3G [53] was also transfected. Alternatively, 293T cells in 10-cm dishes were transfected with 6.5 μg of CHKCG, 4 μg of pCRVI/Gag-PR-Pol, 3 μg of pCR3.1/Rec, and 1.5 μg of VSV-G. At 24 h after transfection, the transfection mixture was replaced with of fresh media containing 5 μM sodium butyrate. To generate HIV-1 (HERV-K_CON_) pseudotypes, 293T cells in 10-cm plates were transfected with 6 μg of HIV-1-GagPol, 6 μg of CSGW, and either 3 μg of pCRVI/HERV-K_CON_ Env or empty pCRVI as a control.

Filtered (0.2 μm) supernatant from HERV-K_CON_(VSV-G)– or HIV-1(HERV-K_CON_)–producing cells was placed onto target cells seeded in 24-well plates along with fresh media in the presence of 5 μg of polybrene/ml for 24 h. Two days after infection, GFP^+^ target cells were quantified either by counting foci microscopically or by FACS analysis. In some experiments, 50 μM AZT was added to the medium at the time of infection.

### Analysis of de novo integrated HERV-K_CON_ proviral DNA.

CHO745 cells were infected with CHKCP-carrying HERV-K_CON_(VSV-G) virus stock and transduced cells selected in 2.5 μg/ml puromycin for approximately 10 d. From the puromycin-resistant population comprising several hundred colonies, four single cell clones were derived by limiting dilution and expanded in culture (approximately 2 wk). Cellular DNA extracted from each clone (Qiagen extraction kit, http://www.qiagen.com) was subjected to PCR analysis using HERV-K_CON_ *gag*-specific primers Gag-S (nucleotides 1236 to 1262, Figure S1) and Gag-AS (nucleotides 1991 to 1946, Figure S1). Additionally, host DNA sequences flanking the integrated CHKCP proviral DNA were cloned using the GenomeWalker kit (Clontech) according to the manufacturer's instructions and PCR primers directed to the HERV-K_CON_ LTRs. Specifically, LTR-AS (GCA AGA GAG ATC AGA TTG TTA CTG TGT CTG) and LTR-S (TAC GAG AAA CAC CCA CAG GTG TGT AGG) oligonucleotides were used to clone sequences flanking the 5′ and 3′ LTRs, respectively. Additional PCR primers, targeting flanking hamster DNA sequences identified via the GenomeWalker approach, were used to authenticate the presence of preintegration sites in uninfected CHO745 cells and integrated provirus in three CHKCP transduced cell clones. In the example (clone No. 1) shown in Figure 4E, the primers Ham-S (GCT ACC CTG AAG ATT TGA GCC AGT GTG C) and Ham-AS (TCT TGC AAG TTG TCC TGT GGC ATG G) were used. For all PCRs, 30 cycles of amplification were completed using 200 ng of cellular DNA, with no DNA, uninfected CHO cell DNA, or human DNA templates analyzed as negative and positive controls, as appropriate.

## Supporting Information

Figure S1 HERV-K_CON_ Proviral Sequence The complete 9,472-nucleotide provial sequence is shown. LTR sequences are underlined. Protein sequences encoded by Gag, PR, Pol, Env, and K-Rev ORFs are also shown.
(77 KB PDF)

## Abbreviations

APOBEC: apolipoprotein B mRNA-editing enzyme, catalytic polypeptide-like
AZT: azidothymidine
CA: capsid
CMV: cytomegalovirus
GFP: green fluorescent protein
HERV: human endogenous retrovirus
HML-2: human mouse mammary tumor virus–like 2
LTR: long terminal repeat
MUV: murine leukemia virus
ORF: open reading frame
TRIM5α: tripartite motif 5α
VLP: virus-like particle
VSV: vesicular stomatitis virus
