# Supplementary material for: Reconstitution of an Infectious Human Endogenous Retrovirus
Source: PLoS Pathog. 2007 Jan 26;3(1):e10. doi: 10.1371/journal.ppat.0030010 (PMC1781480; doi:10.1371/journal.ppat.0030010)
Supplement: Figure S1 — The complete 9,472-nucleotide provial sequence is shown. LTR sequences are underlined. Protein sequences encoded by Gag, PR, Pol, Env, and K-Rev ORFs are also shown. (77 KB PDF) [file ppat.0030010.sg001.pdf]

Figure S1. HERV-K$_{CON}$ proviral sequence.

The complete 9472 nucleotide provial sequence is shown. Long terminal repeat sequences are underlined. Protein sequences encoded by Gag, PR, Pol, Env and K-Rev open reading frames are also shown.

```
                                                            60                                                          120
TGTGGGGAAAAGCAAGAGAGATCAGATTGTTACTGTGTCTGTGTAGAAAGAAGTAGACATAGGAGACTCCATTTTGTTATGTACTAAGAAAAATTCTTCTGCCTTGAGATTCTGTTAATC
                                                           180                                                          240
TATGACCTTACCCCCAACCCCGTGCTCTCTGAAACGTGTGCTGTGTCAACTCAGAGTTGAATGGATTAAGGGCGGTGCAGGATGTGCTTTGTTAAACAGATGCTTGAAGGCAGCATGCTC
                                                           300                                                          360
CTTAAGAGTCATCACCACTCCCTAATCTCAAGTACCCAGGGACACAAAAACTGCGGAAGGCCGCAGGGACCTCTGCCTAGGAAAGCCAGGTATTGTCCAAGGTTTCTCCCCATGTGATAG
                                                           420                                                          480
TCTGAAATATGGCCTCGTGGGAAGGGAAAGACCTGACCGTCCCCCAGCCCGACACCCGTAAAGGGTCTGTGCTGAGGAGGATTAGTAAAAGAGGAAGGAATGCCTCTTGCAGTTGAGACA
                                                           540                                                          600
AGAGGAAGGCATCTGTCTCCTGCCTGTCCCTGGGCAATGGAATGTCTCGGTATAAAACCCGATTGTATGCTCCATCTACTGAGATAGGGAAAAACCGCCTTAGGGCTGGAGGTGGGACCT
                                                           660                                                          720
GCGGGCAGCAATACTGCTTTGTAAAGCACTGAGATGTTTATGTGTATGCATATCTAAAAGCACAGCACTTAATCCTTTACATTGTCTATGATGCAAAGACCTTTGTTCACGTGTTTGTCT
                                                           780                                                          840
GCTGACCCTCTCCCCACAATTGTCTTGTGACCCTGACACATCCCCCTCTTTGAGAAACACCCACAGATGATCAATAAATACTAAGGGAACTCAGAGGCTGGCGGGATCCTCCATATGCTG
                                                           900                                                          960
AACGCTGGTTCCCCGGGTCCCCTTATTTCTTTCTCTATACTTTGTCTCTGTGTCTTTTTCTTTTCCAAATCTCTCGTCCCACCTTACGAGAAACACCCACAGGTGTGTAGGGGCAACCCA
                                                          1020                                                         1080
CCCCTACATCTGGTGCCCAACGTGGAGGCTTTTCTCTAGGGTGAAGGTACGCTCGAGCGTGGTCATTGAGGACAAGTCGACGAGAGATCCCGAGTACGTCTACAGTCAGCCTTACGGTAA
                                                          1140                                                         1200
GCTTGTGCGCTCGGAAGAAGCTAGGGTGATAATGGGGCAAACTAAAAGTAAAATTAAAAGTAAATATGCCTCTTATCTCAGCTTTATTAAAATTCTTTTAAAAAGAGGGGGAGTTAAAGT
                                M  G  Q  T  K  S  K  I  K  S  K  Y  A  S  Y  L  S  F  I  K  I  L  L  K  R  G  G  V  K  V
                                _____________________________________GAG______________________________________
                                                          1260                                                         1320
ATCTACAAAAAATCTAATCAAGCTATTTCAAATAATAGAACAATTTTGCCCATGGTTTCCAGAACAAGGAACTTTAGATCTAAAAGATTGGAAAAGAATTGGTAAGGAACTAAAACAAGC
  S  T  K  N  L  I  K  L  F  Q  I  I  E  Q  F  C  P  W  F  P  E  Q  G  T  L  D  L  K  D  W  K  R  I  G  K  E  L  K  Q  A
___________________________________________________________GAG___________________________________________________________
                                                          1380                                                         1440
AGGTAGGAAGGGTAATATCATTCCACTTACAGTATGGAATGATTGGGCCATTATTAAAGCAGCTTTAGAACCATTTCAAACAGAAGAAGATAGCGTTTCAGTTTCTGATGCCCCTGGAAG
  G  R  K  G  N  I  I  P  L  T  V  W  N  D  W  A  I  I  K  A  A  L  E  P  F  Q  T  E  E  D  S  V  S  V  S  D  A  P  G  S
___________________________________________________________GAG___________________________________________________________
                                                          1500                                                         1560
CTGTATAATAGATTGTAATGAAAACACAAGGAAAAAATCCCAGAAAGAAACGGAAGGTTTACATTGCGAATATGTAGCAGAGCCGGTAATGGCTCAGTCAACGCAAAATGTTGACTATAA
  C  I  I  D  C  N  E  N  T  R  K  K  S  Q  K  E  T  E  G  L  H  C  E  Y  V  A  E  P  V  M  A  Q  S  T  Q  N  V  D  Y  N
___________________________________________________________GAG___________________________________________________________
                                                          1620                                                         1680
TCAATTACAGGAGGTGATATATCCTGAAACGTTAAAATTAGAAGGAAAAGGTCCAGAATTAGTGGGGCCATCAGAGTCTAAACCACGAGGCACAAGTCCTCTTCCAGCAGGTCAGGTGCC
  Q  L  Q  E  V  I  Y  P  E  T  L  K  L  E  G  K  G  P  E  L  V  G  P  S  E  S  K  P  R  G  T  S  P  L  P  A  G  Q  V  P
___________________________________________________________GAG___________________________________________________________
                                                          1740                                                         1800
CGTAACATTACAACCTCAAAAGCAGGTTAAAGAAAATAAGACCCAACCGCCAGTAGCCTATCAATACTGGCCTCCGGCTGAACTTCAGTATCGGCCACCCCCAGAAAGTCAGTATGGATA
  V  T  L  Q  P  Q  K  Q  V  K  E  N  K  T  Q  P  P  V  A  Y  Q  Y  W  P  P  A  E  L  Q  Y  R  P  P  P  E  S  Q  Y  G  Y
___________________________________________________________GAG___________________________________________________________
                                                          1860                                                         1920
TCCAGGAATGCCCCCAGCACCACAGGGCAGGGCGCCATACCCTCAGCCGCCCACTAGGAGACTTAATCCTACGGCACCACCTAGTAGACAGGGTAGTGAATTACATGAAATTATTGATAA
  P  G  M  P  P  A  P  Q  G  R  A  P  Y  P  Q  P  P  T  R  R  L  N  P  T  A  P  P  S  R  Q  G  S  E  L  H  E  I  I  D  K
___________________________________________________________GAG___________________________________________________________
                                                          1980                                                         2040
ATCAAGAAAGGAAGGAGATACTGAGGCATGGCAATTCCCAGTAACGTTAGAACCGATGCCACCTGGAGAAGGAGCCCAAGAGGGAGAGCCTCCCACAGTTGAGGCCAGATACAAGTCTTT
  S  R  K  E  G  D  T  E  A  W  Q  F  P  V  T  L  E  P  M  P  P  G  E  G  A  Q  E  G  E  P  P  T  V  E  A  R  Y  K  S  F
___________________________________________________________GAG___________________________________________________________
                                                          2100                                                         2160
TTCGATAAAAAATGCTAAAAGATATGAAAGAGGGAGTAAAACAGTATGGACCCAACTCCCCTTATATGAGGACATTATTAGATTCCATTGCTCATGGACATAGACTCATTCCTTATGATTG
  S  I  K  M  L  K  D  M  K  E  G  V  K  Q  Y  G  P  N  S  P  Y  M  R  T  L  L  D  S  I  A  H  G  H  R  L  I  P  Y  D  W
___________________________________________________________GAG___________________________________________________________
                                                          2220                                                         2280
GGAGATTCTGGCAAAATCGTCTCTCTCACCCTCTCAATTTTTACAATTTAAGACTTGGTGGATTGATGGGGTACAAGAACAGGTCCGAAGAAATAGGGCTGCCAATCCTCCAGTTAACAT
  E  I  L  A  K  S  S  L  S  P  S  Q  F  L  Q  F  K  T  W  W  I  D  G  V  Q  E  Q  V  R  R  N  R  A  A  N  P  P  V  N  I
___________________________________________________________GAG___________________________________________________________
                                                          2340                                                         2400
AGATGCAGATCAACTATTAGGAATAGGTCAAAATTGGAGTACTATTAGTCAACAAGCATTAATGCAAAATGAGGCCATTGAGCAAGTTAGAGCTATCTGCCTTAGAGCCTGGGAAAAAAT
  D  A  D  Q  L  L  G  I  G  Q  N  W  S  T  I  S  Q  Q  A  L  M  Q  N  E  A  I  E  Q  V  R  A  I  C  L  R  A  W  E  K  I
___________________________________________________________GAG___________________________________________________________
                                                          2460                                                         2520
CCAAGACCCAGGAAGTACCTGCCCCTCATTTAATACAGTAAGACAAGGTTCAAAAGAGCCCTATCCTGATTTTGTGGCAAGGCTCCAAGATGTTGCTCAAAAGTCAATTGCCGATGAAAA
  Q  D  P  G  S  T  C  P  S  F  N  T  V  R  Q  G  S  K  E  P  Y  P  D  F  V  A  R  L  Q  D  V  A  Q  K  S  I  A  D  E  K
___________________________________________________________GAG___________________________________________________________
                                                          2580                                                         2640
AGCCCGTAAGGTCATAGTGGAGTTGATGGCATATGAAAACGCCAATCCTGAGTGTCAATCAGCCATTAAGCCATTAAAAGGAAAGGTTCCTGCAGGATCAGATGTAATCTCAGAATATGT
  A  R  K  V  I  V  E  L  M  A  Y  E  N  A  N  P  E  C  Q  S  A  I  K  P  L  K  G  K  V  P  A  G  S  D  V  I  S  E  Y  V
___________________________________________________________GAG___________________________________________________________
                                                          2700                                                         2760
AAAAGCCTGTGATGGAATCGGAGGAGCTATGCATAAAGCTATGCTTATGGCTCAAGCAATAACAGGAGTTGTTTTAGGAGGACAAGTTAGAACATTTGGAGGAAAATGTTATAATTGTGG
  K  A  C  D  G  I  G  G  A  M  H  K  A  M  L  M  A  Q  A  I  T  G  V  V  L  G  G  Q  V  R  T  F  G  G  K  C  Y  N  C  G
___________________________________________________________GAG___________________________________________________________
                                                          2820                                                         2880
TCAAATTGGTCACTTAAAAAAGAATTGCCCAGTCTTAAACAAACAGAATATAACTATTCAAGCAACTACAACAGGTAGAGAGCCACCTGACTTATGTCCAAGATGTAAAAAAGGAAAACA
  Q  I  G  H  L  K  K  N  C  P  V  L  N  K  Q  N  I  T  I  Q  A  T  T  T  G  R  E  P  P  D  L  C  P  R  C  K  K  G  K  H
___________________________________________________________GAG___________________________________________________________
```

2940 3000
TTGGGCTAGTCAATGTCGTTCTAAATTTGATAAAAAATGGGCAACCATTGTCGGGAAACGAGCAAAGGGGCCAGCCTCAGGCCCCACAACAAACTGGGGCATTCCCAATTCAGCCATTTGT
K W A T I V G K R A K G P A S G P T T N W G I P N S A I C
PROTEASE
W A S Q C R S K F D K N G Q P L S G N E Q R G Q P Q A P Q Q T G A F P I Q P F V
GAG

3060 3120
TCCTCAGGGTTTTCAGGGACAACAACCCCCACTGTCCCAAGTGTTTCAGGGAATAAGCCAGTTACCACAATACAACAATTGTCCCCCGCCACAAGCGGCAGTGCAGCAGTAGATTTATGT
S S G F S G T T T P T V P S V S G N K P V T T I Q Q L S P A T S G S A A V D L C
PROTEASE
P Q G F Q G Q Q P P L S Q V F Q G I S Q L P Q Y N N C P P P Q A A V Q Q *
GAG

3180 3240
ACTATACAAGCAGTCTCTCTGCTTCCAGGGGAGCCCCCACAAAAAATCCCCACAGGGGTATATGGCCCCCTGCCTGAGGGGACTGTAGGACTAATCTTGGGAAGATCAAGTCTAAATCTA
T I Q A V S L L P G E P P Q K I P T G V Y G P L P E G T V G L I L G R S S L N L
PROTEASE

3300 3360
AAAGGAGTTCAAATTCATACTAGTGTGGTTGATTCAGACTATAAAGGCGAAATTCAATTGGTTATTAGCTCTTCAATTCCTTGGAGTGCCAGTCCAGGAGACAGGATTGCTCAATTATTA
K G V Q I H T S V V D S D Y K G E I Q L V I S S S I P W S A S P G D R I A Q L L
PROTEASE

3420 3480
CTCCTGCCATATATTAAGGGTGGAAATAGTGAAATAAAAAGAATAGGAGGGCTTGGAAGCACTGATCCAACAGGAAAGGCTGCATATTGGGCAAGTCAGGTCTCAGAGAACAGACCTGTG
L L P Y I K G G N S E I K R I G G L G S T D P T G K A A Y W A S Q V S E N R P V
PROTEASE

3540 3600
TGTAAGGCCATTATTCAAGGAAAACAGTTTGAAGGGTTGGTAGACACTGGAGCAGATGTCTCTATCATTGCTTTAAATCAGTGGCCAAAAAATTGGCCTAAACAAAAGGCTGTTACAGGA
C K A I I Q G K Q F E G L V D T G A D V S I I A L N Q W P K N W P K Q K A V T G
PROTEASE

3660 3720
CTTGTCGGCATAGGCACAGCCTCAGAAGTGTATCAAAGTACGGAGATTTTACATTGCTTAGGGCCAGATAATCAAGAAAGTACTGTTCAGCCAATGATTACTTCAATTCCTCTTAATCTG
L V G I G T A S E V Y Q S T E I L H C L G P D N Q E S T V Q P M I T S I P L N L
PROTEASE

3780 3840
TGGGGTCGAGATTTATTACAACAATGGGGTGCGGAAATCACCATGCCCGCTCCATTATATAGCCCCACGAGTCAAAAAATCATGACCAAGATGGGATATATACCAGGAAAGGGACTAGGG
W G R D L L Q Q W G A E I T M P A P L Y S P T S Q K I M T K M G Y I P G K G L G
PROTEASE

3900 3960
AAAAATGAAGATGGCATTAAAGTTCCAGTTGAGGCTAAAATAAATCAAGAAAGAGAAGGAATAGGGTATCCTTTTTAGGGGCGGCCACTGTAGAGCCTCCTAAACCCATACCATTAACTT
K N E D G I K V P V E A K I N Q E R E G I G Y P F *
PROTEASE
N K S R K R R N R V S F L G A A T V E P P K P I P L T
POL

4020 4080
GGAAAACAGAAAAACCGGTGTGGGTAAATCAGTGGCCGCTACCAAAACAAAAACTGGAGGCTTTACATTTATTAGCAAATGAACAGTTAGAAAAGGGTCATATTGAGCCTTCGTTCTCAC
W K T E K P V W V N Q W P L P K Q K L E A L H L L A N E Q L E K G H I E P S F S
POL

4140 4200
CTTGGAATTCTCCTGTGTTTGTAATTCAGAAGAAATCAGGCAAATGGCGTATGTTAACTGACTTAAGGGCTGTAAACGCCGTAATTCAACCCATGGGGCCTCTCCAACCCGGGTTGCCCT
P W N S P V F V I Q K K S G K W R M L T D L R A V N A V I Q P M G P L Q P G L P
POL

4260 4320
CTCCGGCCATGATCCCAAAAGATTGGCCTTTAATTATAATTGATCTAAAGGATTGCTTTTTTACCATCCCTCTGGCAGAGCAGGATTGCGAAAAATTTGCCTTTACTATACCAGCCATAA
S P A M I P K D W P L I I I D L K D C F F T I P L A E Q D C E K F A F T I P A I
POL

4380 4440
ATAATAAAGAACCAGCCACCAGGTTTCAGTGGAAAGTGTTACCTCAGGGAATGCTTAATAGTCCAACTATTTGTCAGACTTTTGTAGGTCGAGCTCTTCAACCAGTTAGAGAAAAGTTTT
N N K E P A T R F Q W K V L P Q G M L N S P T I C Q T F V G R A L Q P V R E K F
POL

4500 4560
CAGACTGTTATATTATTCATTATATTGATGATATTTTATGTGCTGCAGAAACGAAAGATAAATTAATTGACTGTTATACATTTCTGCAAGCAGAGGTTGCCAATGCTGGACTGGCAATAG
S D C Y I I H Y I D D I L C A A E T K D K L I D C Y T F L Q A E V A N A G L A I
POL

4620 4680
CATCTGATAAGATCCAAACCTCTACTCCTTTTCATTATTTAGGGATGCAGATAGAAAATAGAAAAATTAAGCCACAAAAAATAGAAATAAGAAAAGACACATTAAAAACACTAAATGATT
A S D K I Q T S T P F H Y L G M Q I E N R K I K P Q K I E I R K D T L K T L N D
POL

4740 4800
TTCAAAAATTACTAGGAGATATTAATTGGATTCGGCCAACTCTAGGCATTCCTACTTATGCCATGTCAAATTTGTTCTCTATCTTAAGAGGAGACTCAGACTTAAATAGTAAAAGAATGT
F Q K L L G D I N W I R P T L G I P T Y A M S N L F S I L R G D S D L N S K R M
POL

4860 4920
TAACCCCAGAGGCAACAAAAGAAATTAAATTAGTGGAAGAAAAAATTCAGTCAGCGCAAATAAATAGAATAGATCCCTTAGCCCCACTCCAACTTTTGATTTTTGCCACTGCACATTCTC
L T P E A T K E I K L V E E K I Q S A Q I N R I D P L A P L Q L L I F A T A H S
POL

4980 5040
CAACAGGCATCATTATTCAAAATACTGATCTTGTGGAGTGGTCATTCCTTCCTCACAGTACAGTTAAGACTTTTACATTGTACTTGGATCAAATAGCTACATTAATCGGTCAGACAAGAT
P T G I I I Q N T D L V E W S F L P H S T V K T F T L Y L D Q I A T L I G Q T R
POL

5100 5160
TACGAATAATAAAATTATGTGGAAATGACCCAGACAAAATAGTTGTCCCTTTAACCAAGGAACAAGTTAGACAAGCCTTTATCAATTCTGGTGCATGGCAGATTGGTCTTGCTAATTTTG
L R I I K L C G N D P D K I V V P L T K E Q V R Q A F I N S G A W Q I G L A N F
POL

5220 5280
TGGGAATTATTGATAATCATTACCCAAAAACAAAGATCTTCCAGTTCTTAAAATTGACTACTTGGATTCTACCTAAAATTACCAGACGTGAACCTTTAGAAAATGCTCTAACAGTATTTA
V G I I D N H Y P K T K I F Q F L K L T T W I L P K I T R R E P L E N A L T V F
POL

5340 5400
CTGATGGTTCCAGCAATGGAAAAGCAGCTTACACAGGGCCGAAAGAACGAGTAATCAAAACTCCATATCAATCGGCTCAAAGAGCAGAGTTGGTTGCAGTCATTACAGTGTTACAAGATT
T D G S S N G K A A Y T G P K E R V I K T P Y Q S A Q R A E L V A V I T V L Q D
POL

5460 5520
TTGACCAACCTATCAATATTATATCAGATTCTGCATATGTAGTACAGGCTACAAGGGATGTTGAGACAGCTCTAATTAAATATAGCATGGATGATCAGTTAAACCAGCTATTCAATTTAT
F D Q P I N I I S D S A Y V V Q A T R D V E T A L I K Y S M D D Q L N Q L F N L
POL

5580 5640
TACAACAAACTGTAAGAAAAAGAAATTTCCCATTTTATATTACTCATATTCGAGCACACACTAATTTACCAGGGCCTTTGACTAAAGCAAATGAACAAGCTGACTTACTGGTATCATCTG
L Q Q T V R K R N F P F Y I T H I R A H T N L P G P L T K A N E Q A D L L V S S
POL

5700 5760
CACTCATAAAAGCACAAGAACTTCATGCTTTGACTCATGTAAATGCAGCAGGATTAAAAAACAAATTTGATGTCACATGGAAACAGGCAAAAGATATTGTACAACATTGCACCCAGTGTC
A L I K A Q E L H A L T H V N A A G L K N K F D V T W K Q A K D I V Q H C T Q C
POL

5820 5880
AAGTCTTACACCTGCCCACTCAAGAGGCAGGAGTTAATCCCAGAGGTCTGTGTCCTAATGCATTATGGCAAATGGATGTCACGCATGTACCTTCATTTGGAAGATTATCATATGTTCATG
Q V L H L P T Q E A G V N P R G L C P N A L W Q M D V T H V P S F G R L S Y V H
POL

5940 6000
TAACAGTTGATACTTATTCACATTTCATATGGGCAACTTGCCAAACAGGAGAAAGTACTTCCCATGTTAAAAAACATTTATTGTCTTGTTTTGCTGTAATGGGAGTTCCAGAAAAAATCA
V T V D T Y S H F I W A T C Q T G E S T S H V K K H L L S C F A V M G V P E K I
POL

6060 6120
AAACTGACAATGGACCAGGATATTGTAGTAAAGCTTTCCAAAAATTCTTAAGTCAGTGGAAAATTTCACATACAACAGGAATTCCTTATAATTCCCAAGGACAGGCCATAGTTGAAAGAA
K T D N G P G Y C S K A F Q K F L S Q W K I S H T T G I P Y N S Q G Q A I V E R
POL

6180 6240
CTAATAGAACACTCAAAACTCAATTAGTTAAACAAAAAGAAGGGGGAGACAGTAAGGAGTGTACCACTCCTCAGATGCAACTTAATCTAGCACTCTATACTTTAAATTTTTTAAACATTT
T N R T L K T Q L V K Q K E G G D S K E C T T P Q M Q L N L A L Y T L N F L N I
POL

6300 6360
ATAGAAATCAGACTACTACTTCTGCAGAACAACATCTTACTGGTAAAAAGAACAGCCCACATGAAGGAAAACTAATTTGGTGGAAAGATAATAAAAATAAGACATGGGAAATAGGGAAGG
Y R N Q T T T S A E Q H L T G K K N S P H E G K L I W W K D N K N K T W E I G K
POL

6420 6480
TGATAACGTGGGGGAGAGGTTTTGCTTGTGTTTCACCAGGAGAAAATCAGCTTCCTGTTTGGATACCCACTAGACATTTGAAGTTCTACAATGAACCCATCGGAGATGCAAAGAAAAGCA
V I T W G R G F A C V S P G E N Q L P V W I P T R H L K F Y N E P I G D A K K S
POL

M N P S E M Q R K A
ENV
M N P S E M Q R K A
K-REV

6540 6600
CCTCCGCGGAGACGGAGACACCGCAATCGAGCACCGTTGACTCACAAGATGAACAAAATGGTGACGTCAGAAGAACAGATGAAGTTGCCATCCACCAAGAAGGCAGAGCCGCCGACTTGG
T S A E T E T P Q S S T V D S Q D E Q N G D V R R T D E V A I H Q E G R A A D L
POL
P P R R R R H R N R A P L T H K M N K M V T S E E Q M K L P S T K K A E P P T W
ENV
P P R R R R H R N R A P L T H K M N K M V T S E E Q M K L P S T K K A E P P T W
K-REV

6660 6720
GCACAACTAAAGAAGCTGACGCAGTTAGCTACAAAATATCTAGAGAACACAAAGGTGACACAAACCCCAGAGAGTATGCTGCTTGCAGCCTTGATGATTGTATCAATGGTGGTAAGTCTC
G T T K E A D A V S Y K I S R E H K G D T N P R E Y A A C S L D D C I N G G K S
POL
A Q L K K L T Q L A T K Y L E N T K V T Q T P E S M L L A A L M I V S M V V S L
ENV
A Q L K K L T Q L A T K Y L E N T K V T Q T P E S M L L A A L M I V S M V
K-REV

6780 6840
CCTATGCCTGCAGGAGCAGCTGCAGCTAACTATACCTACTGGGCCTATGTGCCTTTCCCGCCCTTAATTCGGGCAGTCACATGGATGGATAATCCTATAGAAGTATATGTTAATGATAGT
P Y A C R S S C S *
POL
P M P A G A A A A N Y T Y W A Y V P F P P L I R A V T W M D N P I E V Y V N D S
ENV

6900 6960
GTATGGGTACCTGGCCCCATAGATGATCGCTGCCCTGCCAAACCTGAGGAAGAAGGGATGATGATAAATATTTCCATTGGGTATCGTTATCCTCCTATTTGCCTAGGGAGAGCACCAGGA
V W V P G P I D D R C P A K P E E E G M M I N I S I G Y R Y P P I C L G R A P G
ENV

7020 7080
TGTTTAATGCCTGCAGTCCAAAATTGGTTGGTAGAAGTACCTACTGTCAGTCCCATCAGTAGATTCACTTATCACATGGTAAGCGGGATGTCACTCAGGCCACGGGTAAATTATTTACAA
C L M P A V Q N W L V E V P T V S P I S R F T Y H M V S G M S L R P R V N Y L Q
ENV

7140 7200
GACTTTTCTTATCAAAGATCATTAAAATTTAGACCTAAAGGGAAACCTTGCCCCAAGGAAATTCCCAAAGAATCAAAAAATACAGAAGTTTTAGTTTGGGAAGAATGTGTGGCCAATAGT
D F S Y Q R S L K F R P K G K P C P K E I P K E S K N T E V L V W E E C V A N S
ENV

7260 7320
GCGGTGATATTACAAAACAATGAATTTGGAACTATTATAGATTGGGCACCTCGAGGTCAATTCTACCACAATTGCTCAGGACAAACTCAGTCGTGTCCAAGTGCACAAGTGAGTCCAGCT
A V I L Q N N E F G T I I D W A P R G Q F Y H N C S G Q T Q S C P S A Q V S P A
ENV

7380 7440
GTTGATAGCGACTTAACAGAAAGTTTAGACAAACATAAGCATAAAAAATTGCAGTCTTTCTACCCTTGGGAATGGGGAGAAAAAGGAATCTCTACCCCAAGACCAAAAATAGTAAGTCCT
V D S D L T E S L D K H K H K K L Q S F Y P W E W G E K G I S T P R P K I V S P
ENV

7500 7560
GTTTCTGGTCCTGAACATCCAGAATTATGGAGGCTTACTGTGGCCTCACACCACATTAGAATTTGGTCTGGAAATCAAACTTTAGAAACAAGAGATCGTAAGCCATTTTATACTGTCGAC
V S G P E H P E L W R L T V A S H H I R I W S G N Q T L E T R D R K P F Y T V D
ENV

7620 7680
CTAAATTCCAGTCTAACAGTTCCTTTACAAAGTTGCGTAAAGCCCCCTTATATGCTAGTTGTAGGAAATATAGTTATTAAACCAGACTCCCAGACTATAACCTGTGAAAATTGTAGATTG
L N S S L T V P L Q S C V K P P Y M L V V G N I V I K P D S Q T I T C E N C R L
ENV

7740 7800
CTTACTTGCATTGATTCAACTTTTAATTGGCAACACCGTATTCTGCTGGTGAGAGCAAGAGAGGGCGTGTGGATCCCTGTGTCCATGGACCGACCGTGGGAGGCCTCACCATCCGTCCAT
L T C I D S T F N W Q H R I L L V R A R E G V W I P V S M D R P W E A S P S V H
ENV

7860 7920
ATTTTGACTGAAGTATTAAAAGGTGTTTTAAATAGATCCAAAAGATTCATTTTTACTTTAATTGCAGTGATTATGGGATTAATTGCAGTCACAGCTACGGCTGCTGTAGCAGGAGTTGCA
I L T E V L K G V L N R S K R F I F T L I A V I M G L I A V T A T A A V A G V A
ENV

```
                                                                            7980                                                                            8040
TTGCACTCTTCTGTTCAGTCAGTAAACTTTGTTAATGATTGGCAAAAAAATTCTACAAGATTGTGGAATTCACAATCTAGTATTGATCAAAAATTGGCAAATCAAATTAATGATCTTAGA
 L  H  S  S  V  Q  S  V  N  F  V  N  D  W  Q  K  N  S  T  R  L  W  N  S  Q  S  S  I  D  Q  K  L  A  N  Q  I  N  D  L  R
                                                          ENV
                                                                            8100                                                                            8160
CAAACTGTCATTTGGATGGGAGACAGACTCATGAGCTTAGAACATCGTTTCCAGTTACAATGTGACTGGAATACGTCAGATTTTTGTATTACACCCCAAATTTATAATGAGTCTGAGCAT
 Q  T  V  I  W  M  G  D  R  L  M  S  L  E  H  R  F  Q  L  Q  C  D  W  N  T  S  D  F  C  I  T  P  Q  I  Y  N  E  S  E  H
                                                          ENV
                                                                            8220                                                                            8280
CACTGGGACATGGTTAGACGCCATCTACAGGGAAGAGAAGATAATCTCACTTTAGACATTTCCAAATTAAAAGAACAAATTTTCGAAGCATCAAAAGCCCATTTAAATTTGGTGCCAGGA
 H  W  D  M  V  R  R  H  L  Q  G  R  E  D  N  L  T  L  D  I  S  K  L  K  E  Q  I  F  E  A  S  K  A  H  L  N  L  V  P  G
                                                          ENV
                                                                            8340                                                                            8400
ACTGAGGCAATTGCAGGAGTTGCTGATGGCCTCGCAAATCTTAACCCTGTCACTTGGGTTAAGACCATTGGAAGTACTACGATTATAAATCTCATATTAATCCTTGTGTGCCTGTTTTGT
 T  E  A  I  A  G  V  A  D  G  L  A  N  L  N  P  V  T  W  V  K  T  I  G  S  T  T  I  I  N  L  I  L  I  L  V  C  L  F  C
                                                          ENV

                                                                            8460                                                                            8520
CTGTTGTTAGTCTGCAGGTGTACCCAACAGCTCCGAAGAGACAGCGACCATCGAGAACGGGCCATGATGACGATGGCGGTTTTGTCGAAAAGAAAAGGGGGAAATGTGGGGAAAAGCAAG
 L  L  L  V  C  R  C  T  Q  Q  L  R  R  D  S  D  H  R  E  R  A  M  M  T  M  A  V  L  S  K  R  K  G  G  N  V  G  K  S  K
                                                          ENV
                S  A  G  V  P  N  S  S  E  E  T  A  T  I  E  N  G  P  *
                              K-REV (EXON2)
                                                                            8580                                                                            8640
AGAGATCAGATTGTTACTGTGTCTGTGTAGAAAGAAGTAGACATAGGAGACTCCATTTTGTTATGTACTAAGAAAAATTCTTCTGCCTTGAGATTCTGTTAATCTATGACCTTACCCCCA
 R  D  Q  I  V  T  V  S  V  *
          ENV
                                                                            8700                                                                            8760
ACCCCGTGCTCTCTGAAACGTGTGCTGTGTCAACTCAGAGTTGAATGGATTAAGGGCGGTGCAGGATGTGCTTTGTTAAACAGATGCTTGAAGGCAGCATGCTCCTTAAGAGTCATCACC
                                                                            8820                                                                            8880
ACTCCCTAATCTCAAGTACCCAGGGACACAAAAACTGCGGAAGGCCGCAGGGACCTCTGCCTAGGAAAGCCAGGTATTGTCCAAGGTTTCTCCCCATGTGATAGTCTGAAATATGGCCTC
                                                                            8940                                                                            9000
GTGGGAAGGGAAAGACCTGACCGTCCCCCAGCCCGACACCCGTAAAGGGTCTGTGCTGAGGAGGATTAGTAAAAGAGGAAGGAATGCCTCTTGCAGTTGAGACAAGAGGAAGGCATCTGT
                                                                            9060                                                                            9120
CTCCTGCCTGTCCCTGGGCAATGGAATGTCTCGGTATAAAACCCGATTGTATGCTCCATCTACTGAGATAGGGAAAAACCGCCTTAGGGCTGGAGGTGGGACCTGCGGGCAGCAATACTG
                                                                            9180                                                                            9240
CTTTGTAAAGCACTGAGATGTTTATGTGTATGCATATCTAAAAGCACAGCACTTAATCCTTTACATTGTCTATGATGCAAAGACCTTTGTTCACGTGTTTGTCTGCTGACCCTCTCCCCA
                                                                            9300                                                                            9360
CAATTGTCTTGTGACCCTGACACATCCCCCTCTTTGAGAAACACCCACAGATGATCAATAAATACTAAGGGAACTCAGAGGCTGGCGGGATCCTCCATATGCTGAACGCTGGTTCCCCGG
                                                                            9420
GTCCCCTTATTTCTTTCTCTATACTTTGTCTCTGTGTCTTTTTCTTTTCCAAATCTCTCGTCCCACCTTACGAGAAACACCCACAGGTGTGTAGGGGCAACCCACCCCTACA
```
